# Utility of a Peripheral Intravenous Catheter for Administration of Vasopressors: A Narrative Review

**DOI:** 10.7759/cureus.105205

**Published:** 2026-03-14

**Authors:** Muhammad Hammad Ashraf, Saran Lal Ajai Mokan Dasan, George S Zacharia, Maryam Ali, Fouad Kaddour Hocine, Naqash Mazhar, Diaz Saez Yordanka, Misbahuddin Khaja

**Affiliations:** 1 Internal Medicine, BronxCare Health System, New York City, USA; 2 Internal Medicine, Bronx Care Health System, New York City, USA; 3 Pulmonary Medicine, BronxCare Health System, New York City, USA

**Keywords:** complications, norepinephrine, peripheral intravenous catheter, shock, vasopressor

## Abstract

Vasopressors are the cornerstone of the management of shock, a life-threatening condition characterized by inadequate tissue perfusion, with risks of organ failure and death. Prompt initiation of vasopressors restores hemodynamic stability and prevents shock sequelae. Vasopressors have long been administered via central venous catheters (CVCs) due to concerns about extravasation and tissue injury with peripheral venous administration. However, CVC placement is an invasive, time-consuming procedure requiring specialized personnel and resources, potentially delaying the initiation of life-saving medications. With the 'time to management' being a detrimental factor in shock, there has been a renewed interest in peripheral vasopressor therapy, particularly in emergent or resource-limited settings where complications and delays exceed expectations. Over the last decade, multiple observational studies have demonstrated the safety and efficacy of peripheral vasopressor administration, especially when using large-bore catheters placed in proximal upper extremity veins and under vigilant monitoring. Early initiation of vasopressors via peripheral venous access has been associated with faster attainment of target mean arterial pressure and shorter time to hemodynamic stabilization, without increased mortality or severe complications. Prompt recognition and management of extravasation, including the use of phentolamine, further mitigates risks. Guidelines suggest starting vasopressors peripherally to avoid delays, with a transition to central access if prolonged infusion is anticipated. Institutional protocols, including site selection, catheter type, infusion duration, and escalation criteria, are essential to optimize safety. Further research is needed to refine protocols and address remaining knowledge gaps regarding prolonged use and optimal catheter selection.

## Introduction and background

Shock is a state of decreased tissue perfusion with risk of progression to organ failure and death if not treated at the earliest. Ensuring optimal tissue perfusion while treating the underlying cause is the initial step. Vasopressors play a crucial role in ensuring tissue perfusion in shock [1]. While the diagnostic approach and treatment differ among the four different types of shock (hypovolemic, distributive, cardiogenic, and obstructive shock), vasopressors are invariably used in varied capacities in all these shocks as a temporizing measure. Conventionally, these medications were administered via central venous catheters (CVCs) due to the risks of extravasation and consequent tissue injury when administered peripherally [2-5]. However, CVC placement is an invasive procedure with risks of pneumothorax, bloodstream infections, arterial puncture, and central venous thrombosis [6]. In contrast, peripheral venous cannulation is a less invasive and relatively quick procedure and could be performed in virtually all clinical settings. In an urgent and/or resource-limited setting, securing central venous access can delay the other time-sensitive core measures that have been shown to improve outcomes in shock. The principle of "time is tissue" extends here, as early vasopressor initiation is universally adopted, as delays are associated with worse outcomes [7].

Over the past decade, multiple studies have demonstrated the safety and efficacy of peripheral vasopressor therapy when delivered via appropriately selected veins under careful monitoring [8-10]. This allows early initiation of vasopressors in emergent clinical situations when CVC placement is not immediately feasible. The central-to-peripheral shift was particularly evident during the COVID-19 pandemic, when the use of peripheral access emerged as both a pragmatic and efficacious approach to achieving hemodynamic stability while minimizing procedural risks and healthcare worker exposure [11]. This narrative review explores the utility of peripheral venous lines for vasopressor therapy, summarizing current evidence on their feasibility, rationale, and safety in the management of shock requiring vasoactive agents.

Background and rationale

Central venous catheterization requires trained personnel, ultrasound guidance, sterile technique, and resources that may not be immediately available in all clinical environments. The insertion process can delay the initiation of vasopressors, a factor strongly associated with increased mortality in shock [7,12,13]. In contrast, peripheral intravenous lines are widely accessible, easy to establish, and require minimal resources, allowing immediate commencement of life-saving therapy while central access is being established, if needed [14]. 

The rationale for re-evaluating peripheral vasopressor use arises from several key considerations listed below.

Safety

Accumulating evidence indicates that when vasopressors are infused through appropriately selected veins, the risk of extravasation or tissue injury is remarkably low, around 1%-4% [8,9]. Most reported events are minor and reversible with prompt management [15,16].

Risks of Central Access

CVC insertion, particularly in an emergent setting, poses significant procedural risks, including arterial puncture, pneumothorax, thrombosis, and central line-associated bloodstream infections, all of which could lead to prolonged hospital stays, increased healthcare costs, and higher morbidity [6,17]. The use of peripheral vasopressors can avoid central line requirements by 51% [16].

Timeliness

Early vasopressor initiation is often lifesaving. Initiating vasopressors via a peripheral line can bridge the critical time lag until central venous access is established, improving patient outcomes [12,13]. 

Resource-Limited and Prehospital Settings

In contexts where a central line placement is not timely feasible, peripheral administration protocols allow prompt initiation of vasopressors. This consequently decreases the need for CVC and resultant central line-associated bloodstream infection (CLABSI) [18].

Ethical and Logistical Considerations

Clinicians are obliged to provide the most effective, safe, and timely interventions to the patients. With proper protocols and monitoring, peripheral vasopressor use ensures early hemodynamic stabilization while minimizing procedural risks.

## Review

Methodology and study selection

A focused literature search of PubMed/Medical Literature Analysis and Retrieval System Online (MEDLINE), Excerpta Medica database (Embase), and the Cochrane Library was conducted, supplemented by reference-list screening of key articles and recent guidelines. English-language sources relevant to peripheral IV vasopressor administration in critically ill adults were selected, emphasizing safety, practical protocols, site selection, and complications.

Pharmacologic considerations

A clear understanding of the pharmacology of vasopressors is essential when considering peripheral administration. Vasopressors exert their effects via adrenergic and non-adrenergic pathways to increase the vascular tone and maintain perfusion [19,20]. The most frequently used vasopressors, the mechanism(s) of action, and administration protocols are summarized in Table 1. 

**Table 1:** Common vasopressor agents used in shock and their predominant receptor activity and physiologic effects

Drug	Receptor	Action
Norepinephrine	α1/α2 , β1	Vasoconstriction, inotropic and chronotropic
Epinephrine	α1/α2, β1, β2	Vasoconstriction, inotropic and chronotropic, Vasodilation and catabolism
Dopamine	α1, β1, D	Vasoconstriction, inotropic and chronotropic, dopaminergic
Phenylephrine	α1	Vasoconstriction
Vasopressin	V1, V2	Vasoconstriction, antidiuretic
Metaraminol	α1	Vasoconstriction

Norepinephrine is the first-line drug of choice in septic shock owing to its powerful α1 effects with limited β1 activity. Norepinephrine also poses a lower risk of arrhythmia than its counterpart, dopamine, owing to its limited β1 activity. CVC is conventionally preferred for infusion; however, the drug can be administered via peripheral venous access [21]. Studies, though inconsistent with the recommended maximal dose through the peripheral catheter, suggested that they can be safely administered for up to 48 hours [22]. Peripheral administration is best accomplished through a large-bore cannula inserted into proximal upper extremity veins under regular monitoring [4-16]. Peripheral infusion at higher concentrations or through lower extremity veins or distal upper extremity veins is generally not recommended because of dose-dependent tissue toxicity [23]. 

Vasopressin modulates vascular tone by acting through V1 receptors. It is frequently used as an adjunct to norepinephrine. The Surviving Sepsis Guidelines recommend initiation of vasopressin at a dose of 0.03 units/minute when the norepinephrine dose is in the range of 0.25−0.5 μg/kg/min [20]. The VANISH (Effect of Early Vasopressin vs Norepinephrine on Kidney Failure in Patients With Septic Shock) randomized clinical trial utilized the titrating vasopressin protocol of 0.02 to 0.06 units/minute through a CVC in patients with septic shock [24]. The agent can be peripherally administered when required [25,26].

Epinephrine increases cardiac output and vascular tone via mixed α and β stimulation and increases lactate via β2‑mediated metabolic effects. It is often used as a second-line agent in the treatment of shock [19,20].

Dopamine has a receptor activity that is essentially dose dependent, but it increases the risk of arrhythmias and consequently worsens clinical outcomes as compared to norepinephrine [20,27]. 

Phenylephrine is a pure α₁ agonist. Owing to the lack of β-adrenergic effects, it is preferred in patients with tachyarrhythmia or when β-stimulation is not desired [19].

Metaraminol is an isomer of meta-hydroxynorephedrine and is a potent α1 adrenoceptor agonist and a weak β1 agonist. It is frequently used for the management of septic shock across Australia and Europe, where it is the second most used initial vasopressor, next only to norepinephrine. However, no recommendations were identified in the International Surviving Sepsis Campaign guidelines [28]. Available evidence on peripheral use of epinephrine, dopamine, phenylephrine and metarminol are very limited”.

Peripheral vasopressor therapy: available evidence 

Evidence over the last decade has consistently demonstrated that the clinical efficacy of peripherally administered vasopressors is comparable to that of central venous administration if protocols for appropriate site selection, rigorous monitoring, and dose titration are followed. 

In the CLOVERS (Crystalloid Liberal vs Early Vasopressors in Sepsis) trial, 582 patients received vasopressors, of which 490 patients were initiated via a peripheral line. Also, pressor therapy was continued via peripheral line beyond six hours in 333 patients. This suggests that the peripheral administration of vasopressors is a common clinical practice [29]. A subgroup analysis of patients in the CLOVERS trial who received vasopressors within 24 hours of enrollment but had non-central venous access reported similar 90-day mortality rates for peripheral versus central vasopressor initiation (26.1% vs 37.0%; adjusted odds ratio: 0.67; 95% CI, 0.39-1.16) [30]. The only reported complication of peripheral vasopressor therapy was extravasation, which occurred in three out of 490 patients. Interestingly, CVC placement-related complications were reported in 14 of 322 patients who were in the CLOVERS trial, which included arrhythmias, hematoma formation, and deep vein thrombosis [30]. These data point towards the safety of peripheral vasopressor therapy, at least in controlled settings. These are post hoc analyses; hence, their comparison cannot be validated. 

The VIPCA (Vasopressor Infusion via Peripheral Cannula vs Central Access) trial, a randomized controlled feasibility trial, reported a median time to CVC insertion of 3.3 hours (interquartile range: 1.2-3.7 hours) in the early CVC arm [31]. The days alive and out of the hospital at 30 days were comparable across both groups. Similarly, the length of intensive care unit stay, total hospital stay, 30-day mortality, and complication rates were no different between the study groups. Only one patient had extravasation, while none had tissue injury or necrosis [31]. The data from the VIPCA trial also suggest the feasibility and safety of peripheral vasopressor therapy. 

A prospective cohort study by Yerke et al. concluded that initiating norepinephrine via a peripheral line avoided CVC placement in 51.6% of patients. The overall incidence of extravasation was 5.5%, corresponding to 75.8 events per 1,000 days of PIVC administration (95% CI, 52.8-105.4 events per 1,000 days). Only one patient had severe extravasation. The study followed a predefined protocol with a maximum norepinephrine infusion duration of 48 hours and a maximum dose of 15 μg/min, administered via a 20- or 22-gauge peripheral line inserted into an upper extremity vein above the wrist and below the antecubital fossa, under ultrasound guidance [16]. 

In line with mounting evidence of efficacy and safety, peripheral vasopressor therapy is increasingly adopted by hospitals across the globe. The National Health Service has published regional clinical guidelines on peripheral norepinephrine infusion [23].

Advantages of vasopressors administered through a peripheral compared to a central line have been summarized in Table 2 [17,32].

**Table 2:** Comparison of peripheral versus central venous administration of vasopressors in critically ill adults *antecubital, basilic, cephalic; CLABSI: central line-associated bloodstream infection

Factors	Peripheral	Central
Time to initiation	Immediate; few minutes	Delayed: > 30–60 minutes
Site	Large proximal peripheral vein*(32)	Internal jugular, subclavian, femoral
Infection risk (CLABSI)	Very low	Moderate to high (17)
Extravasation risk	1%–4%; often mild; <0.1% severe (32)	-
Procedural complications	Minimal	Pneumothorax, arterial puncture, bleeding, thrombosis (6)
Duration of safe use	Short term: 24 -48 hours	Long term
Resource requirement	Minimal	Trained personnel, ultrasound

Peripheral norepinephrine infusion is unanimously justified for short durations, at low to moderate doses [15,21]. However, the definition of short duration or low-to-moderate doses is rather vague. Published literature has adopted varying dosing protocols. Table 3 summarizes the dosing protocols evaluated or recommended.

**Table 3:** Recommended or studied peripheral vasopressor infusion protocols from the published clinical studies and clinical guidelines. min: minute; hr: hour

	Concentration	Dose	Maximum duration
Norepinephrine			
Yerke et al. [15,16]		Max:15 μg/min	48 hr
Cárdenas-García et al. [15]	8 or 16 mg/250 ml		72 hr
Zichichi et al. [33]	4 mg/250 ml	Start: 0.02 µg/kg/min; Max: 0.5 µg/kg/min	24 hr
Chen et al. [34]	16 µg/mL	Max: 25 µg/min	24 hr
NHS Worcestershire [35]	16 μg/ml	Start: 0.05 µg/kg/min	
NHS Royal Alexandra [23]	4 mg/250 ml	Start: 0.05 µg/kg/min; Max: 0.2 µg/kg/min	24 hr
Western Australia (WA) Country Health [36]	4 mg/500 ml		12 hr
Intensive Care Society [37]	16 µg/mL	Start: 0.05 µg/kg/min	
Phenylephrine			
Cárdenas-García et al. [15]	80 or 160 mg/250 ml		72 hr
Zichichi et al. [33]	40 mg/250 ml	Start: 0.25 µg/kg/min; Max: 4.5 µg/kg/min	24 hr
NHS Worcestershire [35]	100 μg/ml	Start: 10-40 ml/hr	
Chen et al. [34]	100 µg/mL	Max: 10.8 mg/hr	24 hr
Intensive Care Society [37]	100 µg/mL	Max: 10.8 mg/hr	
Dopamine			
Cárdenas-García et al. [15]	400 or 800 mg/250 ml		72 hr
WA Country Health [36]	200 mg/500 ml		12 hr
Epinephrine			
Zichichi et al. [33]	4 mg/250 ml	Start: 0.02 µg/kg/min; Max: 0.25 µg/kg/min	24 hr
NHS Worcestershire [35]	16 μg/ml	Start: 0.05 µg/kg/min	
WA Country Health [36,38]	3 mg/500 ml		12 hr
Chen et al. [34]	16 µg/mL	25 µg/min	24 hr
Intensive Care Society [37]	16 µg/mL	Start: 0.05 µg/kg/min	
Metaraminol			
NHS Worcestershire [35]	0.5 mg/ml	1-20 ml/hr	
Chen et al. [34]	0.5 mg/ml	Max: 10 mg/hr	24 hr
Vasopressin			
Evans et al. [20]		0.03 units/min	
NHS Highland	0.4 U/mL	Start: 0.03 U/min; Range: 0.01-0.04 U/min	
McCurry et al. [25]		Range: 0.01-0.08 U/min; Median: 0.03 units/min	7 hr

Clinicians should have predetermined escalation criteria to convert to central access, for example, (a) Need for higher dose [2,20], (b) Anticipated requirement for prolonged vasopressor support [15], and or (c) Poor peripheral site integrity, like recurrent infiltration, or swelling, are all potential considerations for escalating to centrally administered vasopressors. Most studies recommend establishing institutional protocols to determine the type of catheter to be employed, titration milestones, vasopressor concentrations, and the frequency of local site monitoring [8,9,14].

Complications

Commonly reported complications include phlebitis, bruises, hematoma formation, and cellulitis [39]. Prompt recognition markedly reduces the severity of complications and tissue injury. Extravasation is the primary local complication of concern when considering peripheral administration of vasopressors. The incidence of extravasation varies widely across the literature, ranging from <1 to up to 4% [30]. The incidence of extravasation increases with the duration of peripheral infusion [34]. Early signs include swelling, pain, blanching, pallor, coolness, or decreased capillary refill at the infusion site. In case of extravasation, immediate cessation of the infusion and, if feasible, aspiration of residual drug from the catheter is recommended [10]. The affected limb should be elevated, and warm compresses should be applied. Preferred pharmacotherapy for catecholamine extravasation is phentolamine, a non-selective, competitive ɑ-adrenergic antagonist. Phentolamine is to be subcutaneously infiltrated into the area within 12 hours of extravasation. The recommended dose is 10 to 20 mg in 10 ml of saline within 12 hours [40]. The initial dose is administered into the interstitial catheter prior to removal. Another 10 ml is divided into five injections of 2 ml each on the edge of the swelling. Can be repeated in 60 mins. Owing to its vasodilatory effects, it helps mitigate ischemic injury when administered promptly [41]. Topical nitroglycerin (2% ointment) local application every eight hours until symptom resolution and subcutaneous β₂-agonists (terbutaline-1 mg in 10 ml, can be repeated in 15 minutes) have also been used in case reports/series where phentolamine is unavailable [15,42]. Institutional extravasation protocols should specify dosages, dilution, routes, and escalation steps. 

Tissue ischemia/necrosis is the most dreaded complication of peripheral vasopressor therapy. However, many prospective studies reported no cases of tissue ischemia or necrosis with peripheral vasopressor therapy [30,31]. The anecdotal evidence lacks consistency to provide unbiased recommendations and assessments.

Site selection

The preferred vein for peripheral vasopressor therapy should be of large diameter, allowing higher flow rates and rapid drug dilution, located away from joints to reduce mechanical dislodgement, and easy to monitor visually for early signs of infiltration or phlebitis [34]. The upper extremity veins, such as the basilic or cephalic veins, are considered the safest and most effective locations for peripheral vasopressor administration [32]. Other acceptable sites are the mid-forearm veins or the external jugular vein when upper-limb access is not possible [32]. The sites to avoid include hand, wrist, and foot veins, as they are of small caliber and carry a high risk of extravasation and tissue injury [15,43,44]. Lower extremity veins are not preferred owing to poor venous return, higher infection risk, and slower flow rates. Veins adjacent to joints are prone to increased risk of catheter kinking or dislodgement [34,45]. The infusion needs frequent monitoring; published literature recommends two-hourly assessment for any evidence of extravasation [46]. 

Catheter gauge and length

The length and gauge (G) of the catheter directly influence the safety and efficacy of peripheral vasopressor infusion. A larger bore catheter (18-20 G) allows better flow and reduces the risk of occlusion and infiltration [2]. The length of the catheter will enable it to remain stable within the vein, reducing the risk of dislodgement and extravasation [47]. Long peripheral or midline catheters are particularly helpful for vasopressor therapy lasting 12-24 hours or more. Midlines are approximately 8-15 cm long and provide central-line-like stability with lower invasive risk [47]. 

Ultrasound guidance

**Figure 1:**
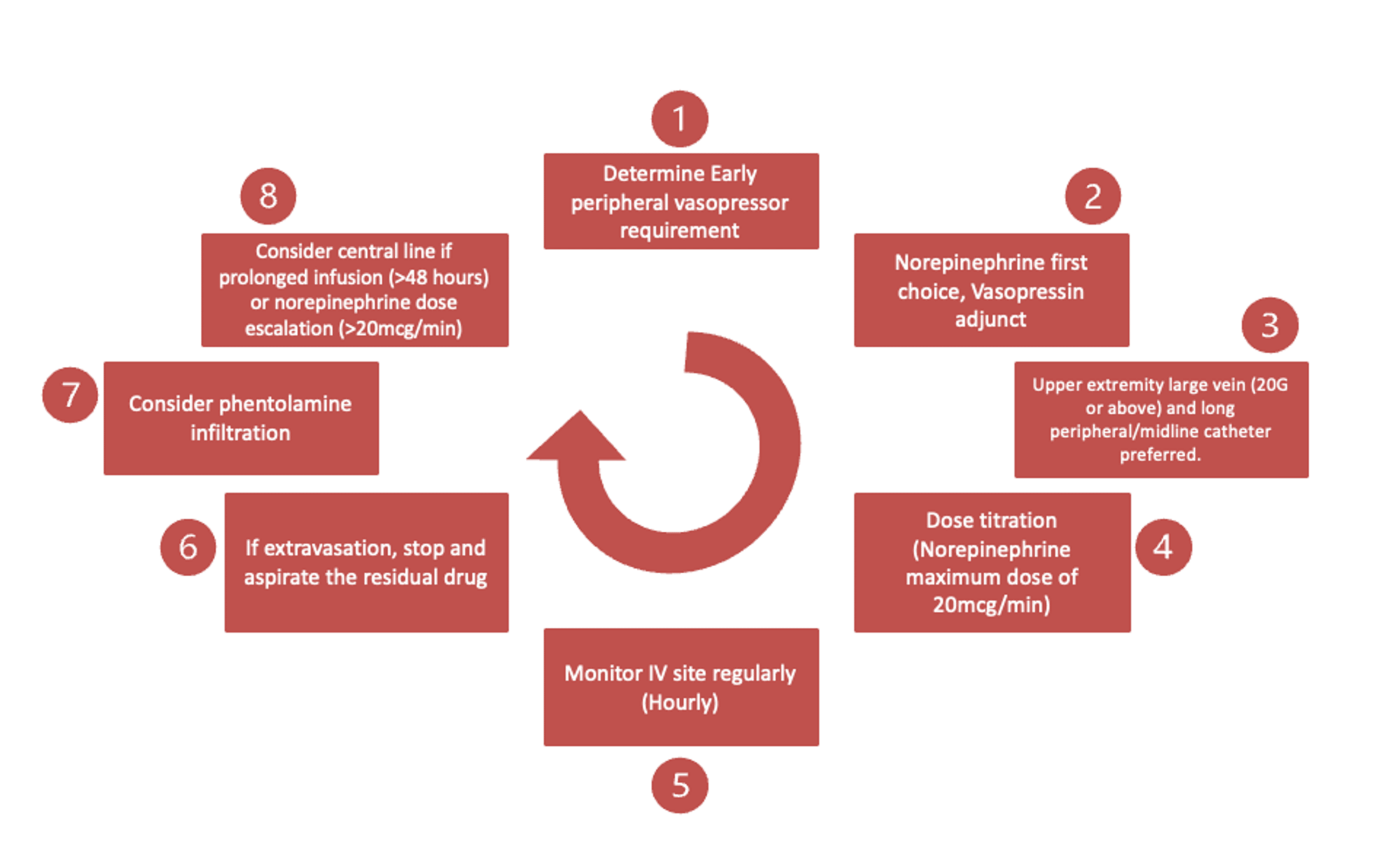
Summary algorithm for peripheral intravenous vasopressor administration and extravasation management This figure was created by the authors using Microsoft PowerPoint (Microsoft Corp., Redmond, WA, USA).

The use of ultrasound guidance significantly improves first-pass success rates and reduces the number of complications [48]. It helps accurate visualization of vein depth and size, and helps avoid tortuous or small veins. Published literature recommends a vein diameter of at least 4 mm, as determined by ultrasound [46]. Additionally, ultrasound guidance ensures the catheter tip is placed in the largest possible segment of the vessel. Overall, ultrasound guidance should be the practice of choice, especially when dealing with deeper brachial and basilic veins. Figure 1 summarises the algorithm for initiation, administration, and management of peripherally administered vasopressors.

Limitations of available evidence

The CLOVERS trial is one of the earliest studies that has shown safety associated with peripheral vasopressor use. The CLOVERS trial’s primary clinical outcome was mortality associated with early vasopressor therapy vs. liberal crystalloid therapy in shock patients, and as such, this study is not powered to detect complications associated with peripheral vasopressors. The VIPCA trial is a randomized controlled feasibility trial comparing central vs. peripherally administered vasopressors, which suggests that clinical outcomes could be similar between the two groups. Though this study suffers from poor protocol adherence and a limited sample size of 20 patients in each group. Multiple other studies followed, comparing the outcomes and complications associated with peripheral vasopressors. A Swedish multicenter retrospective cohort study of norepinephrine infused through a midline catheter reported 2.5% major complications, of which only 0.2% were extravasations [49]. Although no prospective studies have evaluated the upper limits of peripheral vasopressors in terms of dosage and duration, both are notable factors in complications, as demonstrated in a retrospective study by Jung et al. [50]. The SPOTLESS (Safety of PrehOspiTaL pEripheral vaSopreSsors) study is a retrospective study of prehospital vasopressor use, which reported 1% extravasation of vasopressors [51]. Another retrospective study involving peripheral vasopressors administered in the emergency department demonstrated a 4.5% extravasation risk [52]. Shyu et al., in their retrospective analysis comparing central vs peripheral vasopressors, noted only 0.8% extravasation in the peripheral vasopressor group, with a statistically significant decrease in length of hospital stay [53]. A prospective observational study evaluating peripheral vasopressors in emergency department patients reported two patients with extravasation out of 55 [54]. A prospective study done in Ethiopia demonstrated an extravasation risk of 1.2% [55]. All the evidence summarized in this narrative review is mostly derived from observational studies or post hoc limited analysis from randomized controlled studies done for other clinical questions. As such, these recommendations cannot be generalized until more prospective randomized controlled trials analyzing the clinical outcomes in a diverse clinical setting are made available. No grading of evidence has been done in this review owing to the narrative nature of the discussion.

## Conclusions

It is feasible to reliably and safely infuse vasopressors through a peripheral venous access, especially in an emergency setting for early resuscitation. To achieve this goal, critical care teams should emphasize the use of large-bore proximal vessels, diluted infusions, infusion pumps with built-in alarms that detect possible occlusion or infiltration, frequent site inspection, and easy access to reversal agents such as phentolamine. Institutions adopting such protocols have consistently demonstrated reduced central line use, expedited initiation of vasopressor therapy in shock with minimal extravasation injuries, and clinical outcomes comparable to those with centrally administered vasopressors. Most of the safety outcomes have been extrapolated from the trials done for other clinical questions, and hence, they are underpowered to detect safety outcomes. Hence, these recommendations cannot be extended to universal/non-emergent clinical settings. With locally adapted guidelines and appropriate safety protocols, this previously unconventional approach to the management of emergent shock could improve patient outcomes and decrease central line-related costs.
